# Structural Remodeling of the Lumbar Spine Following Non-Surgical Spinal Decompression: An Automated MRI Study

**DOI:** 10.3390/jfmk11040390

**Published:** 2026-09-29

**Authors:** Jacob A. Connolly, Nathan Molinier, Kenneth A. Weber, Julien Cohen-Adad, Nathan D. Schilaty

**Affiliations:** 1 Neurosurgery, Brain & Spine, University of South Florida, 13220 USF Laurel Drive MDF 2108, Tampa, FL 33620, USA; connolly45@usf.edu; 2 Center for Neuromusculoskeletal Research, University of South Florida, Tampa, FL 33620, USA; 3 Morsani College of Medicine, University of South Florida, Tampa, FL 33602, USA; 4 Genie Electrique, Polytechnique Montreal (Ecole Polytechnique), 2900 Edouard-Montpetit Bld, Montreal, QC H3T 1J4, Canada; nathan.molinier@polymtl.ca (N.M.); julien.cohen-adad@polymtl.ca (J.C.-A.); 5 Mila–Quebec AI Institute, Montreal, QC H2S 3H1, Canada; 6 Anesthesiology, Perioperative and Pain Medicine, Stanford University School of Medicine, 1070 Arastradero Road, Suite 200, Palo Alto, CA 94304, USA; kenweber@stanford.edu; 7 Medical Engineering, University of South Florida, Tampa, FL 33620, USA

**Keywords:** non-surgical spinal decompression, magnetic resonance imaging, *SpineReport*, low back pain

## Abstract

**Background and Objectives**: To investigate MRI changes using *SpineReport* (r20260928) following non-surgical spinal decompression (NSSD) in patients with chronic low back pain (cLBP). We conducted a retrospective review of patients treated with NSSD who obtained MRI within 6 months of treatment initiation. **Methods**: Thirty-six subjects (20 F, 16 M), with a mean age of 55.9 ± 16.4 years, were treated at the USF Health Chiropractic Clinic, Department of Neurosurgery, Brain & Spine. NSSD using DRX9000^®^ (Excite Medical, Tampa, FL, USA) was utilized, with a median of 20 (20, 25) treatments. Treatment levels included L3 (*n* = 2), L4 (*n* = 18), and L5 (*n* = 16). Sagittal T2-weighted images were processed using *TotalSpineSeg* (r20251124) for automated segmentation, with *SpineReport* used to quantify disc, vertebrae, spinal canal, and intervertebral foramen metrics. **Results**: Mean pain scores improved by 3.4 ± 1.8 from 5.6 ± 1.6 (*p* < 0.001), while Oswestry Disability Index scores improved by 10.8 ± 9.1 from 17.3 ± 8.0 (*p* < 0.001). At the treated level, spinal canal cross-sectional area increased from 170.9 to 175.8 mm^2^ (2.9%, *p* = 0.040), and right–left diameter increased from 16.0 to 16.3 mm (1.9%, *p* = 0.025). Disc volume decreased from 7762.8 to 7449.5 mm^3^ (−4.0%, *p* = 0.046). No significant changes were observed in disc thickness, DHI, intervertebral foramen area, or vertebral measurements (*p* > 0.05). **Conclusions**: *SpineReport* enabled automated quantification of key spinal structures. NSSD was associated with increased spinal canal dimensions and improved symptoms. Further research is needed to determine the persistence and clinical significance of these structural changes.

## 1. Introduction

Low back pain (LBP) is among the most common musculoskeletal disorders worldwide and remains a leading cause of disability, impaired physical function, and reduced quality of life [1,2]. Approximately 80–85% of individuals experience LBP during their lifetime, with nearly one in five developing chronic symptoms (persistent pain >3 months, muscle stiffness, reduced range of motion) [3]. Consequently, LBP places a substantial burden on healthcare systems and society, contributing substantially to the estimated $980 billion spent annually on musculoskeletal disorders in the United States [2,4].

LBP is commonly categorized according to symptom duration as acute (<6 weeks), subacute (6–12 weeks), or chronic (>12 weeks), and by etiology as specific or non-specific [5]. While specific LBP is attributable to an identifiable pathology such as fracture, infection, malignancy, or disc herniation, approximately 80–90% of cases are classified as non-specific because no definitive structural cause can be identified [6,7]. Clinical practice guidelines recommend patient education, exercise therapy, and behavioral interventions as first-line treatment for chronic non-specific LBP [8]. However, many patients continue to experience persistent pain despite conservative care, while alternative treatments such as opioid therapy and spinal fusion are associated with limited long-term benefit, increased complication rates, and substantial healthcare costs [9,10,11,12,13,14,15,16].

These limitations have motivated interest in non-invasive treatment strategies, including non-surgical spinal decompression (NSSD). NSSD uses computer-controlled traction to apply cyclic distraction forces to the lumbar spine with the goal of reducing mechanical loading on the intervertebral discs. The DRX9000^®^ (Excite Medical, Tampa, FL, USA) is one such device developed to treat discogenic LBP. Previous studies have demonstrated improvements in pain and disability following NSSD, accompanied by increases in intervertebral disc height and reductions in disc herniation size [17,18,19,20]. However, the anatomical changes that occur throughout the lumbar spine following treatment remain poorly understood.

Magnetic resonance imaging (MRI) is the preferred imaging modality for evaluating LBP because it provides high-resolution visualization of intervertebral discs, neural structures, paraspinal muscles, and spinal ligaments. Although MRI has become integral to the assessment of spinal pathology, conventional radiologic interpretation remains largely qualitative and is subject to considerable inter-reader variability, potentially influencing diagnosis and treatment decisions [21]. Automated image analysis platforms, such as *SpineReport* [22], address this limitation by providing standardized, quantitative measurements of spinal anatomy, including disc morphology, spinal canal dimensions, and neural foraminal size. The utility of automated MRI analysis for quantifying anatomical changes following NSSD has not been established.

The purpose of this study was to evaluate whether NSSD treatment is associated with short-term changes in intervertebral disc, spinal canal, neural foramen, and vertebral morphologic metrics in individuals with chronic LBP (cLBP) using automated MRI analysis techniques. We hypothesized that automated MRI analysis would detect measurable changes in spinal canal morphology, intervertebral disc, and neural foraminal anatomy after NSSD treatment.

## 2. Materials and Methods

This study followed all ethical guidelines for retrospective record review with oversight of the University of South Florida Institutional Review Board (STUDY004354). MRI images were obtained from USF Neurosurgery, Brain & Spine Chiropractic Division (Tampa, FL, USA), Wakeman Chiropractic (Daytona Beach, FL, USA), and Spinal Health & Rehab (Punta Gorda, FL, USA).

Subjects were eligible for analysis if the baseline MRI was obtained within six months before treatment initiation and the follow-up MRI within six months after treatment completion, with no specified maximum interval between the two MRI scans.

All patients received NSSD treatment with the DRX9000^®^. Patients were placed in a supine position with the knees flexed to 90°, and a harness was secured around the waist. The initial treatment force was set at 50% of the patient’s body weight (BW) minus 10 lb and progressively increased to 50% BW plus 10 lb. The rate of force progression is individualized for each patient based on comfort.

Additionally, patients completed the numeric pain rating scale (NPRS 0–10; 0 = no pain, 10 = worst imaginable pain) and the Oswestry Disability Index (ODI 0–50; 0 = no disability, 50 = completely disabled) before and after NSSD treatment [23].

DICOM images were de-identified using a custom Python (v3.10) script and then converted into NIFTI format using the *dcm2niix* (v1.0.20241211) conversion tool [24]. T2-weighted sagittal images were then processed through *TotalSpineSeg* (r20251124) [25] to generate segmentations. After segmentation, *SpineReport* (r20260928) [22] was used to extract the following metrics (Table 1). Spinal canal dimensions were defined for each vertebral level by referencing the inferior border of the disc to the superior border of the overlying vertebral body. These values were then averaged to provide a discrete value for each vertebral level. As the vertebral anatomy was not expected to change during treatment, ICC_3,k_ was calculated for vertebral metrics (AP Diameter, Median Thickness, Volume) to determine stability of vertebral metrics over time. ICC calculations were performed in SPSS (v31) (IBM Corp, Armonk, NY, USA).

A Mann–Whitney U test was used to determine differences in age, height, and weight between male and female participants at baseline. A matched pairs analysis was performed to elucidate differences between pre- and post-NSSD measures within subjects. A Bowker’s Test was performed for changes in Pfirrmann grading. Additionally, Spearman’s ρ correlation was used to determine the relationship between MRI metrics and patient-reported outcome measures (PROMs). Metrics are reported as change from baseline (Δ = post–pre). Calculations were performed in JMP Pro 19 (SAS Inc, Cary, NC, USA).

## 3. Results

### 3.1. Demographics

Sixty-six T2-weighted sagittal MRIs were obtained from the three clinical sites. After applying the inclusion criteria, 30 were excluded due to MRIs being outside the designated window of time. Thus, this study included 36 participants (20 F, 16 M) who underwent both pre- and post-treatment MRI of the lumbar spine. Magnetic field strength ranged from 0.25 to 3.0 T. The median interslice spacing was 5.0 mm (IQR, 4.6–5.0 mm), slice thickness was 4.0 mm (IQR, 4.0–4.0 mm), with a median echo time of 100 ms (IQR, 99–108 ms) and repetition time of 3375 ms (IQR, 2587–3800 ms).

The cohort had a mean age of 55.2 ± 16.4 years, a mean height of 170.0 ± 11.9 cm, and a mean weight of 83.4 ± 25.4 kg. Demographic breakdown by sex is detailed in Table 2. Treatment with the DRX9000^®^ targeted the L3 (*n* = 2), L4 (*n* = 18), and L5 (*n* = 16) levels, with a median of 20 sessions (range 19–29) of 28 min in length. The median interval between MRIs was 141.5 days (range 75–424).

**Figure 1:**
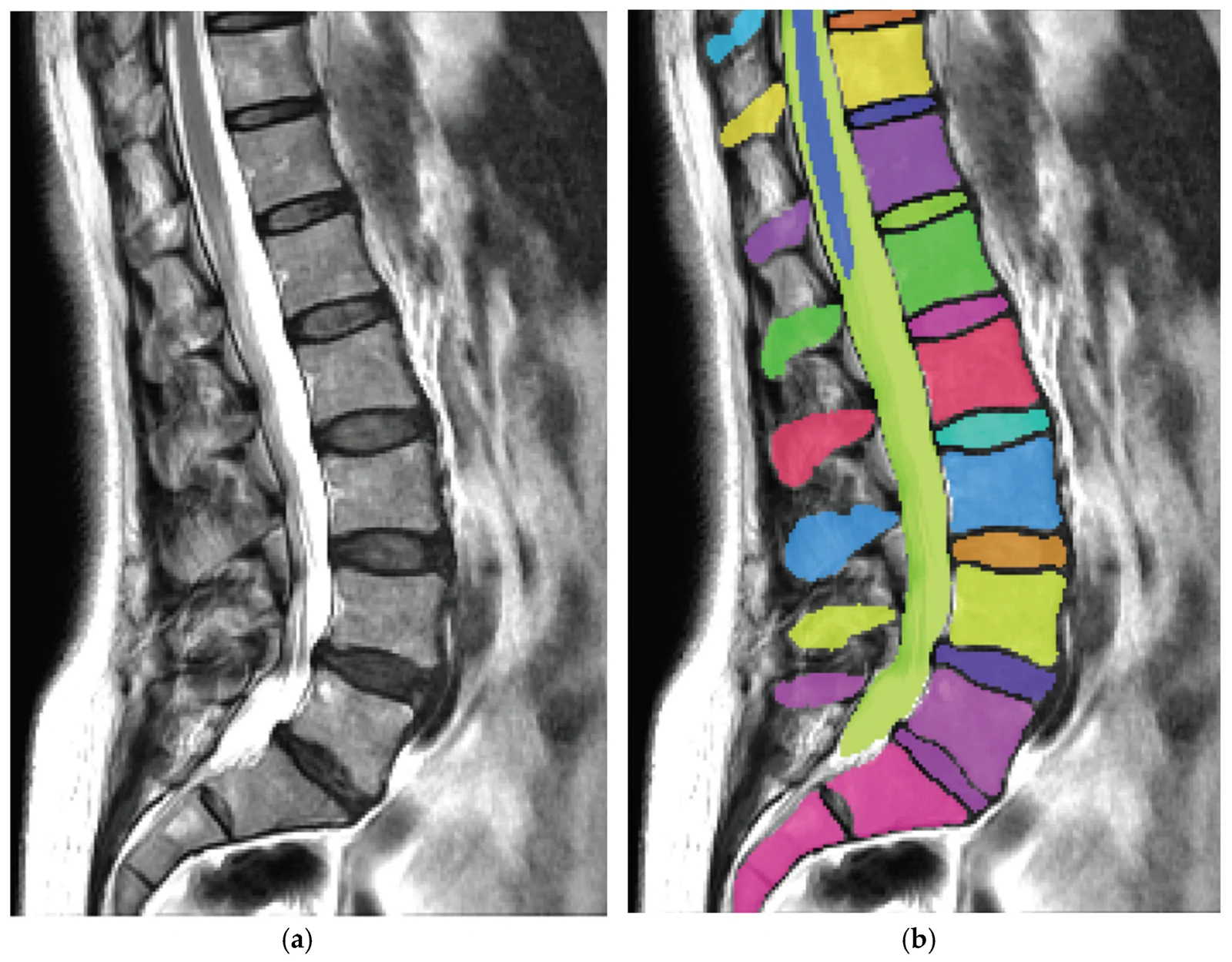
(**a**) Representative T2w sagittal image that was inputted into *TotalSpineSeg*. (**b**) Representative output from *TotalSpineSeg*. The colors distinguish the anatomical structures.

**Figure 2:**
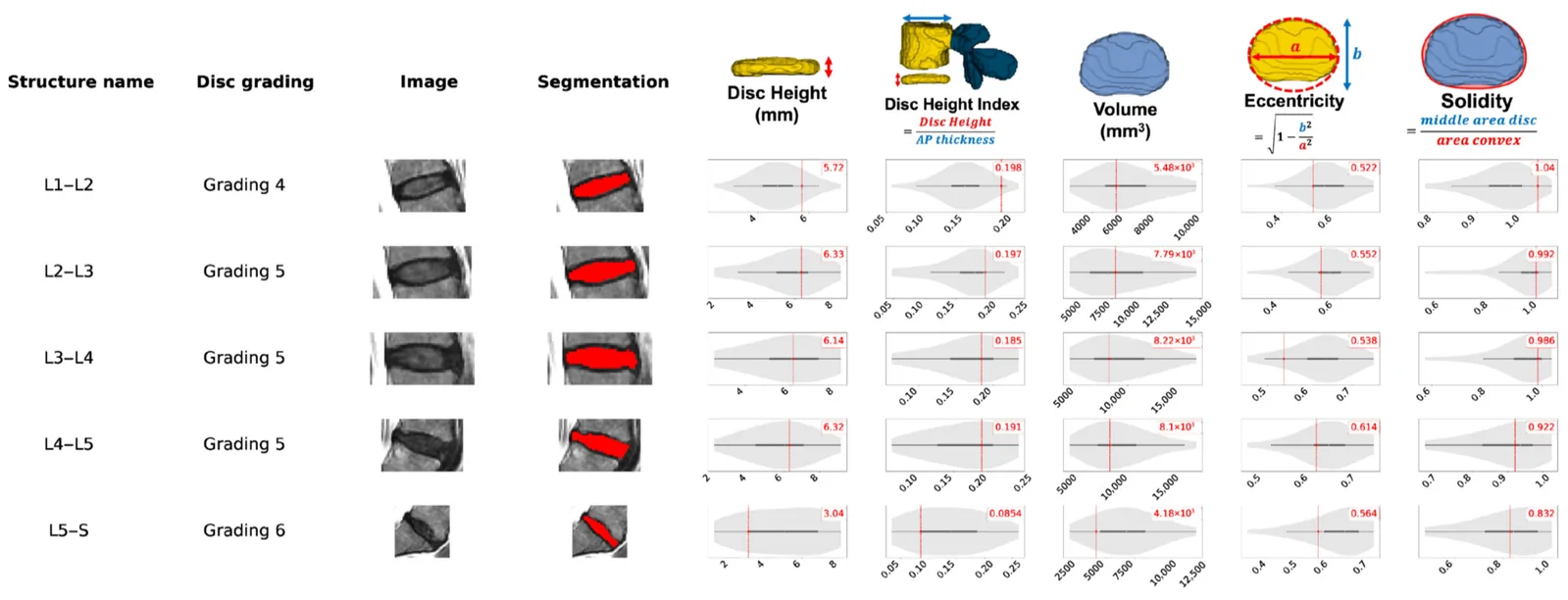
Representative report of disc metrics output by *SpineReport*.

Figure 1a shows an example input image, and Figure 1b shows the *TotalSpineSeg* output. Figure 2 is a representative report of disc metrics generated by *SpineReport*.

### 3.2. Post-Treatment

#### 3.2.1. PROMs

Improvements were observed in PROMs following treatment. Mean pain scores improved by 3.4 ± 1.8 points from a baseline of 5.6 ± 1.6 (*p* < 0.001). Follow-up ODI was available in 20 participants (11 F, 9 M). ODI scores improved by 10.8 ± 9.1 points from a baseline of 17.3 ± 8.0 (*p* < 0.001).

#### 3.2.2. Disc Measures

At the treated vertebral level, disc volume decreased from 7762.8 mm^3^ to 7449.6 mm^3^ (−4.0%, *p* = 0.046). There were no significant changes in median thickness, DHI, solidity, or eccentricity (*p* > 0.05). When examining only the subgroup treated at L5, median thickness decreased from 4.4 mm to 3.9 mm (−10.3%, *p* = 0.009), disc volume decreased from 6223.2 mm^3^ to 5665.0 mm^3^ (−9.9%, *p* = 0.018), and DHI decreased from 0.117 to 0.105 (−11.4%, *p* = 0.006). No changes were observed in the subgroups treated at L3 or L4.

When correlated with PROMs, there was a correlation between ΔDisc Median Thickness and ΔODI (ρ = −0.4878, *p* = 0.029). Additionally, in those treated at L4, there was a correlation between ΔNucleus Median Thickness and ΔODI (ρ = −0.7333, *p* = 0.025).

#### 3.2.3. Pfirrmann Grade

At the level of treatment, five participants showed a reduction in their modified Pfirrmann grade after treatment, two of whom decreased by two levels. Six subjects had an increase in Pfirrmann grade by one level. The remaining 25 showed no change after treatment. The Bowker’s Test revealed no change in Pfirrmann grade among participants (*p* = 0.996).

#### 3.2.4. Canal Measures

**Figure 3:**
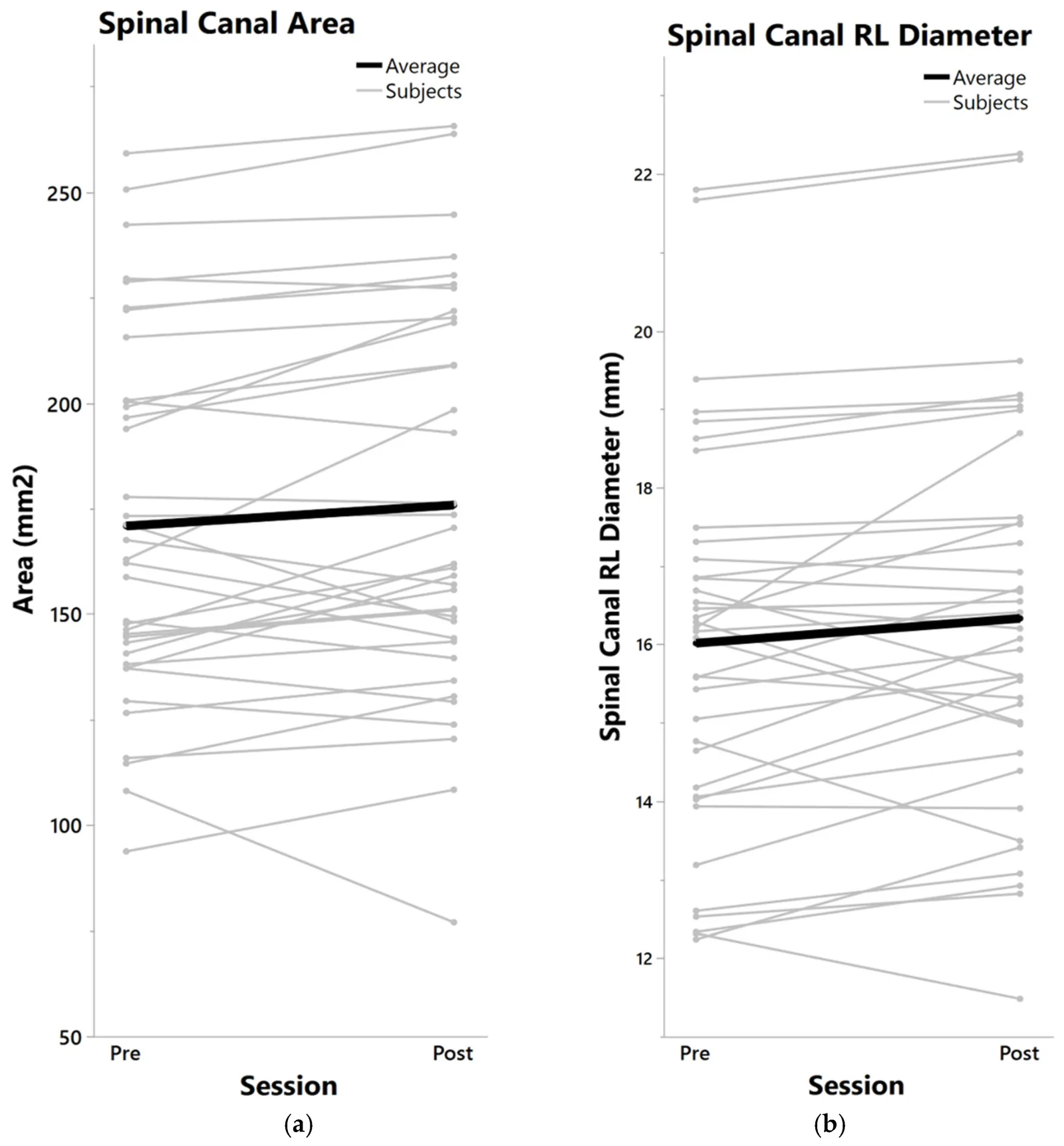
(**a**) Matched pairs analysis of spinal canal cross-sectional area at the level treated (Post–Pre). Average = +4.97 mm. (**b**) Matched pairs analysis of the right–left diameter of the spinal canal at level treated (Post–Pre). Average = +0.32 mm.

The average area of the spinal canal at the level treated increased from 170.9 mm^2^ to 175.8 mm^2^ (2.9%, *p* = 0.040) (Figure 3a). Right–left (RL) diameter of the canal increased from 16.0 mm to 16.3 mm (1.9%, *p* = 0.025) (Figure 3b). No changes were seen in the anterior–posterior (AP) diameter of the spinal canal or in spinal canal eccentricity and solidity.

#### 3.2.5. Foramen Measures

No changes in foramen area were observed for either the right or left side (*p* > 0.05) or in RL asymmetry.

#### 3.2.6. Vertebrae Measures

No changes in vertebrae metrics (AP Thickness, Median Thickness, or Volume) were observed after treatment (*p* > 0.05). AP Thickness had an ICC of 0.983 (0.966, 0.991), Median Thickness = 0.975 (0.950, 0.987), and Volume = 0.996 (0.992 to 0.998).

## 4. Discussion

The purpose of this study was to evaluate whether NSSD treatment is associated with short-term changes in intervertebral disc, spinal canal, neural foramen, and vertebral morphologic metrics in individuals with cLBP. Following NSSD treatment, participants demonstrated substantial improvements in PROMs, including a mean pain reduction of 3.4 points (60.7%), and a 10.8-point (62.4%) improvement in the ODI. These values exceed the established minimal clinically important differences (MCIDs) for pain (2–2.8 points) and ODI (8.5–10 points) [26,27,28].

These clinical improvements were accompanied by measurable structural changes on MRI. Disc volume decreased by approximately 4%, and within the L5 subgroup, reductions in median disc thickness and disc height index (DHI) were observed, suggesting remodeling of the intervertebral disc. Furthermore, the mean spinal canal cross-sectional area at the treated level increased by 4.9 mm^2^ (2.9%), while the right–left (RL) canal diameter increased by 0.3 mm (1.9%), indicating increased canal dimensions following treatment. Notably, changes in disc median thickness and nucleus median thickness were strongly correlated with improvements in PROMs, suggesting that alterations in disc morphology may represent one mechanism contributing to improved pain and function after NSSD.

*SpineReport* has previously demonstrated strong associations between spinal canal anterior–posterior (AP) diameter and cross-sectional area (ρ = 0.59 and 0.56, respectively) and radiologist-graded lumbar central canal stenosis [22]. Additionally, sagittal T1- and T2-weighted MRI scans acquired during the same imaging session demonstrated excellent agreement for disc measurements, with intraclass correlation coefficients (ICCs) ranging from 0.95 to 0.98 for volumetric and thickness-related metrics. Measurements of spinal canal area and AP diameter also showed excellent reproducibility, with ICCs ranging from 0.97 to 0.98. These objective measurements enable reproducible longitudinal assessment of structural changes and have the potential to improve evaluation of treatment response.

Although decreases in disc height can often be considered unfavorable [29,30], the present findings may instead represent adaptive structural remodeling rather than progressive disc degeneration; however, the mechanism underlying the observed reductions in disc volume and thickness remains speculative. One potential explanation is that NSSD treatment alters intradiscal fluid distribution through cyclical loading and unloading of the intended motion segment(s). Previous biomechanical studies have demonstrated that intervertebral discs exhibit dynamic fluid exchange in response to mechanical loading, with changes in hydration status occurring over relatively short time periods [31,32,33,34]. Therefore, reductions in disc dimensions may reflect temporary fluid redistribution rather than structural tissue loss. Alternatively, the observed changes may represent normalization of abnormal disc geometry (e.g., disc herniation) associated with chronic loading and antalgic adaptive patterns that have altered due to improved patient function and decreased LBP. Future studies incorporating quantitative MRI techniques capable of assessing disc hydration and composition will be necessary to better characterize these responses.

In addition to changes in disc morphology, increases in spinal canal area and diameter were observed following treatment. These findings indicate that remodeling of spinal structures may extend beyond the intervertebral disc and may be associated with increased space within the spinal canal. Although the absolute changes in spinal canal dimensions were modest, even small alterations in available neural space may be clinically relevant in individuals with preexisting narrowing from a disc lesion. Because symptoms associated with spinal stenosis and nerve root compression may arise from a combination of mechanical and inflammatory factors, we posit that relatively small anatomical changes could potentially reduce neural irritation and contribute to symptom improvement. While the precise mechanisms underlying these changes remain unclear, they may contribute to the improvements in pain and physical function observed following treatment.

Aside from the changes observed in the disc and spinal canal measures, vertebral measurements served as an internal control, as meaningful changes in vertebral morphology were not expected over the relatively short follow-up period (median 4.7 months). We observed excellent repeatability in vertebrae metrics with ICCs ranging from 0.975 to 0.996. The stability of these vertebral measurements provides validity and increases confidence that the changes observed in disc and canal parameters reflect true anatomical differences rather than variability introduced by imaging acquisition parameters or differences in field of view between imaging sessions.

The improvements observed in PROMs are consistent with previous studies evaluating NSSD treatment [17,20], further supporting the association of NSSD therapy with reductions in pain and disability. To explore the relationship between clinical improvement and structural changes, correlations were examined between changes in PROMs and disc morphology. A significant negative correlation was observed between ΔODI and ΔMedian Disc Thickness and ΔNucleus Median Thickness. Because greater improvements in disability are represented by more negative ΔODI values, this finding indicates that participants who experienced the largest reductions in disability tended to demonstrate increases, or smaller decreases, in median disc thickness following treatment.

Contrary to our hypothesis, neural foraminal dimensions did not demonstrate significant changes following treatment. This may indicate that the observed decompressive effects were localized primarily to the central canal rather than the neural foramina. Alternatively, foraminal changes may be smaller and more variable than changes in central canal dimensions, requiring larger sample sizes to detect reliably. The assessment of these relatively small structures may also have been limited by the resolution of clinical MRI scans, partial volume effects, and variability in imaging hardware and acquisition protocols across sites. Future studies using standardized, higher-resolution imaging and larger cohorts may help determine whether specific patient subgroups exhibit greater foraminal responses.

While current clinical guidelines recommend against routine MRI for patients with acute, subacute, or chronic LBP [35], MRI is still obtained in approximately 16–21% of cases [36]. Given the high lifetime prevalence of LBP, this corresponds to millions of lumbar MRI examinations performed annually. Although MRI plays an important role when clinically indicated, interpretation remains inherently subjective and can vary substantially between radiologists and imaging centers. Herzog et al. demonstrated this variability by sending the same patient to 10 different imaging centers, reporting a global Fleiss’ κ of 0.20 ± 0.06, which indicated poor agreement among radiology reports [21,37]. The authors concluded that the imaging center at which a patient undergoes MRI may directly influence the reported diagnosis, subsequent treatment decisions, and ultimately clinical outcomes. Given the substantial variability in conventional radiology reporting, there is a need for standardized, objective image analysis to complement clinical decision-making. Automated MRI analysis tools such as *SpineReport* [22] provide reproducible quantitative measurements of clinically relevant spinal morphology while substantially reducing the time required compared with traditional manual measurements.

In the present study, *SpineReport* detected changes in spinal canal area, spinal canal diameter, and disc morphology following NSSD treatment, whereas vertebral morphologic metrics remained unchanged, supporting the specificity of the observed soft tissue changes. Although structural MRI findings have historically demonstrated weak or inconsistent relationships with pain and disability, significant associations between changes in disc morphology and improvements in ODI scores suggest that quantitative imaging metrics may capture clinically meaningful structural adaptations. While these associations do not establish causality, they support the feasibility of automated quantitative MRI for longitudinal assessment of treatment response and highlight its potential to identify imaging biomarkers that may guide individualized patient management. Future studies with larger cohorts are needed to determine whether these imaging-derived metrics can reliably predict clinical outcomes following NSSD.

Previous investigations of spinal decompression therapies have largely focused on subjective clinical outcomes, whereas objective imaging evidence of structural change remains limited. The present findings therefore contribute important imaging-based evidence demonstrating measurable alterations in spinal canal and disc morphology following treatment. While direct comparison between studies is challenging because of differences in imaging methods, treatment protocols, and patient populations, the current results suggest that structural adaptations may accompany the clinical improvements previously reported with NSSD interventions.

### Limitations

Several limitations should be considered when interpreting these findings. This study relied on clinically acquired T2-weighted MRI scans rather than a standardized research imaging protocol. Although imaging data were obtained from multiple radiology centers, all participants included in the final analysis received treatment at a single site (USF Health). Additionally, MRI examinations were performed across different imaging facilities; consequently, acquisition parameters varied between scans, including differences in slice thickness and interslice spacing. The retrospective design precludes causal inference regarding the relationship between NSSD treatment and the observed structural changes, and the absence of a control group makes it difficult to distinguish treatment-related effects from natural variation over time. The relatively modest sample size may have limited statistical power to detect smaller morphological changes and may restrict broader applicability of the findings. While morphologic analyses included 36 participants (20 F, 16 M), follow-up disability outcomes were available for only 20 individuals (11 F, 9 M), further reducing statistical power for analyses involving clinical outcomes. MRI examinations were performed in the supine position, which may not fully reflect spinal morphology under functional loading conditions, but this is a common issue for most clinical scans. Finally, although automated segmentation provides standardized and reproducible measurements, errors related to image quality, segmentation accuracy, and variations in acquisition parameters remain possible.

As this was an exploratory study, additional research is warranted to better characterize the long-term structural effects of NSSD on the intervertebral discs, spinal canal, neural foramina, and vertebral anatomy. Due to the retrospective nature of this study and the lack of a control group, we are unable to determine a causal relationship between NSSD treatment and the improvement in PROMs and MRI metrics we observed. Furthermore, all statistical tests were exploratory, and no corrections for multiple comparisons were applied. Prospective longitudinal studies with larger, multi-center cohorts, standardized imaging protocols, appropriate comparison groups, and comprehensive clinical outcome assessments are needed to confirm these preliminary findings and determine their generalizability across diverse patient populations.

## 5. Conclusions

Treatment of the lumbar spine with NSSD was associated with increases in spinal canal cross-sectional area and right–left canal diameter, accompanied by a minimal reduction in disc volume. These structural changes occurred alongside improvements in patient-reported pain and disability, suggesting that NSSD may promote short-term remodeling of the intervertebral disc and surrounding spinal anatomy. While the underlying biological mechanisms remain to be fully elucidated, the observed morphological changes may contribute to the clinical improvements reported following treatment. Although the relatively small sample size and heterogeneity in MRI acquisition parameters limit the generalizability of these findings, the results provide convincing preliminary evidence that NSSD may produce clinically meaningful structural and functional changes in individuals with cLBP. Additionally, this study demonstrates the utility of automated, open-source spine analysis tools, including *TotalSpineSeg* and *SpineReport*, for objectively quantifying longitudinal changes in spinal morphology, supporting their potential applications in future clinical research and patient monitoring.

## Figures and Tables

**Table 1:** Metrics output by *SpineReport*. (DHI: ratio of median thickness of disc to AP thickness of overlying vertebrae; AP-RL: anterior–posterior/right–left; AP-SI: anterior–posterior/superior–inferior; RL-SI: right–left/superior–inferior).

Intervertebral Disc	Spinal Canal	Vertebral Foramen	Vertebrae
Median Thickness Volume Disc Height Index (DHI) Disc Solidity Disc Eccentricity ○ AP-RL ○ AP-SI ○ RL-SI Nucleus pulposus solidity Nucleus pulposus eccentricity ○ AP-RL ○ AP-SI ○ RL-SI Modified Pfirrmann Grade	Area AP Diameter RL Diameter Eccentricity Solidity	Right Foramen cross-sectional area Left Foramen cross-sectional area R/L Asymmetry	AP Thickness Median Thickness Volume

**Table 2:** Patient demographics. Significance between groups is denoted by *** for *p* < 0.001.

	Female	Male	*p*-Value
Age (yrs)	54.4 ± 17.5	56.1 ± 15.4	0.873
Height (cm)	161.8 ± 7.7	180.2 ± 7.4	<0.001 ***
Weight (kg)	67.4 ± 13.5	103.3 ± 22.6	<0.001 ***

## Data Availability

Data are available upon request.

## Abbreviations

The following abbreviations are used in this manuscript:

LBP	Low Back Pain
MRI	Magnetic Resonance Imaging
NSSD	Non-Surgical Spinal Decompression
PROMs	Patient Reported Outcome Metrics
